# Impact of military training stress on hormone response and recovery

**DOI:** 10.1371/journal.pone.0265121

**Published:** 2022-03-10

**Authors:** Jamie L. Tait, Jace R. Drain, Sean L. Corrigan, Jeremy M. Drake, Luana C. Main

**Affiliations:** 1 Institute for Physical Activity and Nutrition, School of Exercise and Nutrition Sciences, Deakin University, Geelong, Australia; 2 Defence Science and Technology Group, Fisherman’s Bend, Australia; 3 School of Exercise and Nutrition Sciences, Deakin University, Geelong, Victoria, Australia; University of Pisa, ITALY

## Abstract

**Objectives:**

Military personnel are required to train and operate in challenging multi-stressor environments, which can affect hormonal levels, and subsequently compromise performance and recovery. The aims of this project were to 1) assess the impact of an eight-day military training exercise on salivary cortisol and testosterone, 2) track the recovery of these hormones during a period of reduced training.

**Methods:**

This was a prospective study whereby 30 soldiers (n = 27 men, n = 3 women) undergoing the Australian Army combat engineer ‘Initial Employment Training’ course were recruited and tracked over a 16-day study period which included an eight-day military training exercise. Non-stimulated saliva samples were collected at waking, 30 min post waking, and bedtime on days 1, 5, 9, 13, 15; measures of subjective load were collected on the same days. Sleep was measured continuously via actigraphy, across four sequential study periods; 1) baseline (PRE: days 1–4), 2) field training with total sleep deprivation (EX-FIELD: days 5–8), 3) training at simulated base camp with sleep restriction (EX-BASE: days 9–12), and 4) a three-day recovery period (REC: days 13–15).

**Results:**

Morning cortisol concentrations were lower following EX-FIELD (p<0.05) compared to the end of REC. Training in the field diminished testosterone concentrations (p<0.05), but levels recovered within four days. Bedtime testosterone/cortisol ratios decreased following EX-FIELD and did not return to pre-training levels.

**Conclusions:**

The sensitivity of testosterone levels and the testosterone/cortisol ratio to the period of field training suggests they may be useful indicators of a soldier’s state of physiological strain, or capacity, however inter-individual differences in response to a multi-stressor environment need to be considered.

## Introduction

Military personnel are required to work in challenging multi-stressor environments that typically involve a combination of physical, psychological, and cognitive demands, sleep loss, and caloric restriction [1,2]. Exposure to these stressors across military training and operations needs to be balanced with adequate recovery to maintain health and performance [1,3]. Inadequate recovery and an accumulation of training and non-training stress can lead to fatigue, short-term decrements in performance [3,4], and maladaptive responses including negative psychological symptoms and hormonal alterations [5]. These symptoms are also associated with overtraining and have been reported in military training environments [6–8]. For military organisations, insufficient recovery and excessive training loads can increase injury rates [9], and lead to decreased operational capability and increased health care costs [9].

Reliable and actionable metrics of training loads and recovery in military training and operations are yet to be established. Training-induced changes in circulating levels of cortisol and testosterone, and the testosterone/cortisol ratio (T:C; representing the balance between anabolic and catabolic activity), have shown potential in non-military populations as markers of excessive training stress, physiological strain and inadequate recovery [5,10,11]. These hormones are also sensitive to various forms of military training stress involving prolonged physical activity and sleep loss for periods lasting 10 days to 11 weeks, with cortisol concentrations increasing during and following training [12–14], and total and free testosterone concentrations exhibiting decreases [15–17]. Further, decreases in the free T:C ratio have been associated with overtraining following training [6].

Sleep deprivation has also been associated with endocrine changes [8,18,19], particularly in the absence of cardiorespiratory strain [20]. Complete or partial overnight sleep deprivation affects the circadian release of cortisol, leading to elevated cortisol levels in the morning, afternoon and evening following sleep loss [21–23]. Similarly, the cortisol awakening response (CAR; the spike in cortisol levels 30–45 min post-morning awakening) is absent or attenuated after complete overnight sleep deprivation because of the absence of the sleep-to-wake transition [21,24], while partial sleep loss has mixed effects on CAR [22]. Collectively, disruption between anabolic and catabolic hormones may extend recovery times [25,26], suppress immunity [27], and diminish physical and cognitive performance [14,28,29]. Training-induced changes in hormones could therefore be used to monitor training strain and recovery in military personnel, to indicate positive training adaptations [7] and minimise injury risk.

Despite reported changes in hormonal levels following sustained military operations and training, the time course of their recovery post-training is unclear. Training-induced elevations in cortisol levels can persist for up to 2 weeks following military training [13,14,30], while three recovery days may also promote a return to resting levels [31]. Similarly, reductions of testosterone have remained up to two weeks post-training [14,30], while four days of recovery may be sufficient to restore basal levels [13,15,31]. These inconsistencies may result from differences in the length and type of training, stressors present, or activities performed during recovery. To better understand hormonal responses to military training, the current investigation examined the eight-day field training exercise that concludes the Australian Army Combat Engineer Initial Employment Training (IET) course. This multi-stressor exercise incorporates physically and cognitively demanding occupational tasks, combined with periods of sleep deprivation and restriction. An assessment of hormonal levels during and following this exercise may reveal prognostic markers of training-induced physiological strain, whereas establishing time frames of recovery for these markers may contribute to soldier management. Therefore, the aims were to determine the impact of training-induced fatigue and reduced sleep on testosterone and cortisol levels, and track the recovery of these levels in periods of reduced training load and partially restored sleep.

## Materials and methods

### Participants and design

**Fig 1:**
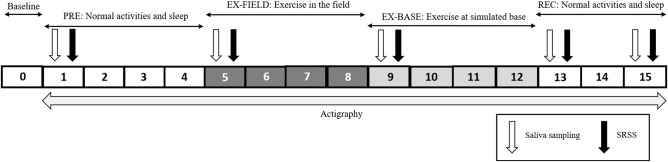
Outline of the 16-day data collection period, comprising four distinct periods. SRSS: Short recovery stress scale.

Data collection occurred as a part of a wider study designed to investigate the impact of an 8-day capstone assessment task for Initial Employment Training, on cognition and heart rate variability (HRV), which was embedded within a 16-day study period. For this study, a conservative power analysis, using HRV, the ‘noisiest’ variable of interest, was undertaken and revealed a minimum sample size of 19 was required [32]. To account for attrition of up to 30% with statistical power of α = 0.80, a sample size of 25 was sought. Participants were Australian Army soldiers undertaking the 18-week Combat Engineer IET course at the School of Military Engineering (Holsworthy Barracks, NSW). Our 16-day study was divided into four distinct periods which included the final eight-day capstone assessment exercise for this course (Fig 1). Salivary hormone levels and subjective stress and recovery data were collected on days 1, 5, 9, 13 and 15. Data was collected in soldiers’ dormitories on days 1,13,15, remaining data was collected in the field (days 5 and 9). Sleep and physical activity were captured continuously. This study was approved by the Department of Defence and Veteran’s Affairs Human Research Ethics Committee (Protocol 021–17). Informed written and verbal consent was obtained from each participant.

### Protocol

Soldiers were briefed on the investigation on day 0, after which voluntary written informed consent and baseline anthropometric data were collected. Following day 0, soldiers underwent four days (days 1 to 4) of normal training and activities (PRE), after which they began the eight-day assessment exercise (EX). Soldiers spent four days living in the field (EX-FIELD; days 5–8) which involved digging trenches, patrols, simulated minefield breaches and responding to contacts. Soldiers were given minimal sleep opportunities, including at least one night of total sleep deprivation on the first night of the EX, with the potential for one other 24h period of sleep deprivation during the EX. After EX-FIELD, soldiers transitioned to a simulated base (EX-BASE) where they simulated repelling enemy attack and completed pickets, with disrupted, restricted sleep taken opportunistically during the day and night (days 9–12). Soldiers then completed the three-day recovery (REC; days 13–15) data collection phase, where they engaged in light activities (e.g., cleaning, returning equipment) with normal sleep opportunities (Fig 1).

### Anthropometry and body composition

Height was measured to the nearest 0.1 cm and body weight was measured to the nearest 0.01 kg using standard techniques (stadiometer and a metric scale respectively). Body mass index (BMI) was calculated [weight (kg)/height (m^2^)].

### Salivary cortisol and testosterone

Salivary hormone levels are highly correlated with those in blood [33,34] and as saliva sampling is a less invasive collection method that reduces participant burden, it is preferable for data collection in remote locations [35]. To control for diurnal rhythm participants provided non-stimulated saliva samples upon waking (0600h in the lines, field), 30 minutes post-waking and immediately before bed/normal time of sleep (2200h). Saliva samples were collected using Salivettes (Sarstedt, Germany), a swab-based sampling device [36]. Participants were instructed to roll the swabs around in their mouth for 2 min. Participants were not available to provide a bedtime saliva sample on day 15. Participants were fasted for waking and 30 minutes post-waking samples, and were instructed to refrain from eating or drinking before bedtime sampling and avoid alcohol for at least 12 hours prior to sample collection. Samples were initially stored at Holsworthy base in a -20°C freezer, then transported to a laboratory where they were stored (in a -80°C freezer) until analysis. Biomarkers were analysed in duplicate. Saliva cortisol and testosterone were analysed using ELISA kits (IBL International, Hamburg, Germany; RE52611 and RE52631) as per manufacturers’ recommendations. All analyses recorded an intra-assay % coefficient of variation (%CV) of <10% and an inter-assay %CV of <20%.

### Sleep and step counts

Activity monitors (Actigraph GT9-X, Pensacola, FL) were used to assess sleep quantity and step counts, worn on the non-dominant wrist 24-h per day during the data collection period, unless contact with water was likely, or when charging the units. Upon completion of data collection, raw activity counts, measured in one-minute epochs, were uploaded using a device-specific interface unit and analysed using the manufacturer’s propriety software (Actilife v6.13). The Cole–Kripke sleep-wake detection algorithm was used to distinguish sleep and wake periods [37]. This algorithm has demonstrated 88% agreement (i.e., percentage of sleep and wake epochs correctly identified) when compared with polysomnography [37]. The participants wore the device for the entire duration of the study except for when they were removed for charging on day 5. Participants occasionally undertook daytime naps, and night-time sleep was often broken in order to perform night duties (e.g., picket/sentry duty). Therefore, to analyse 24-h sleep patterns, sleep duration was aggregated from the end of one night’s main sleep until the end of the next night’s main sleep. Where total sleep deprivation occurred across a 24 h period, sleep duration was entered as ‘0’ in data spreadsheets. External workload was captured with the ActiGraph set at 30 Hz using 60-second epochs. Non-wear time was determined as more than three hours of consecutive ‘0’ total acceleration per day, except days 1 and 5, where eight hours was used to account for downloading and charging. The 3-hour non-wear threshold is more stringent than the standard minimum 10-hour/day total wear time often used in free living adults [38].

### Training stress and recovery

The Short Recovery Stress Scale assesses both recovery and stress state of an individual at the time of surveying [39]. Participants answered eight questions upon waking at data collection periods. Recovery-related questions assessed Physical Performance Capability, Mental Performance Capability, Emotional Balance, and Overall Recovery; stress-related questions assessed Muscular Stress, Lack of Activation, Negative Emotional State, and Overall Stress. Participants indicated the extent to which each statement applied to them at the time of surveying, ranging from 0 (does not apply at all) to 6 (fully applies). Individual item ratings from each subscale were summed to form composite scores for recovery and stress, higher scores indicating greater recovery and stress respectively.

### Statistical analyses

All statistical analyses were conducted using SPSS v.26.0 (SPSS Inc., Armonk, NY). Most data and their residuals were normal prior to analysis; testosterone/cortisol ratio was log transformed. Effects of the training exercise on hormonal levels, external and subjective load, and the trajectory of these variables during recovery, were analysed using linear mixed models with random effects, adjusting for participant variability and sex. To compare the effect of training on both waking and 30 min post-waking hormonal levels, fixed effects were established as condition (from the three sampling timepoints; waking, 30 min post-waking, bedtime), and time (as a repeated measure), to determine within-group variability over the course of the study period. All models utilised the autoregressive heterogenous (ARH1) covariance matrix, while Bonferroni post hoc estimations adjusted for multiple comparisons. To reduce the influence of inter-individual variation in hormone responses, concentrations of cortisol and testosterone at days 5, 9, 13 and 15 were normalised to the first sampling point for each participant (i.e. day 1 of PRE). Testosterone/cortisol ratio was calculated as: testosterone/cortisol concentration, after converting testosterone concentrations to ng/mL. CAR was calculated as (concentration at 30 min post-awakening–concentration at awakening/concentration at awakening) x 100. All data are presented as means ± SEM with 95% CI, except demographic data (mean ± SD). Significance level was set at p<0.05.

## Results

### Participants

In total, 62 soldiers consented to participate in the broader study designed to investigate cognition and training load. From these, a subset of 30 participants (27 men and 3 women) provided saliva samples at all time points, and were included in the current study; aged 22.7 ± 3.8 years (mean ± SD); body mass: 80.5 ± 12.2 kg, height: 1.78 ± 0.1 m; and body mass index 25.3 ± 3.4 kg·m^-2^. There were no differences in the physical characteristics between the recruited cohort and the subset.

### Salivary cortisol and testosterone

#### Cortisol

**Fig 2:**
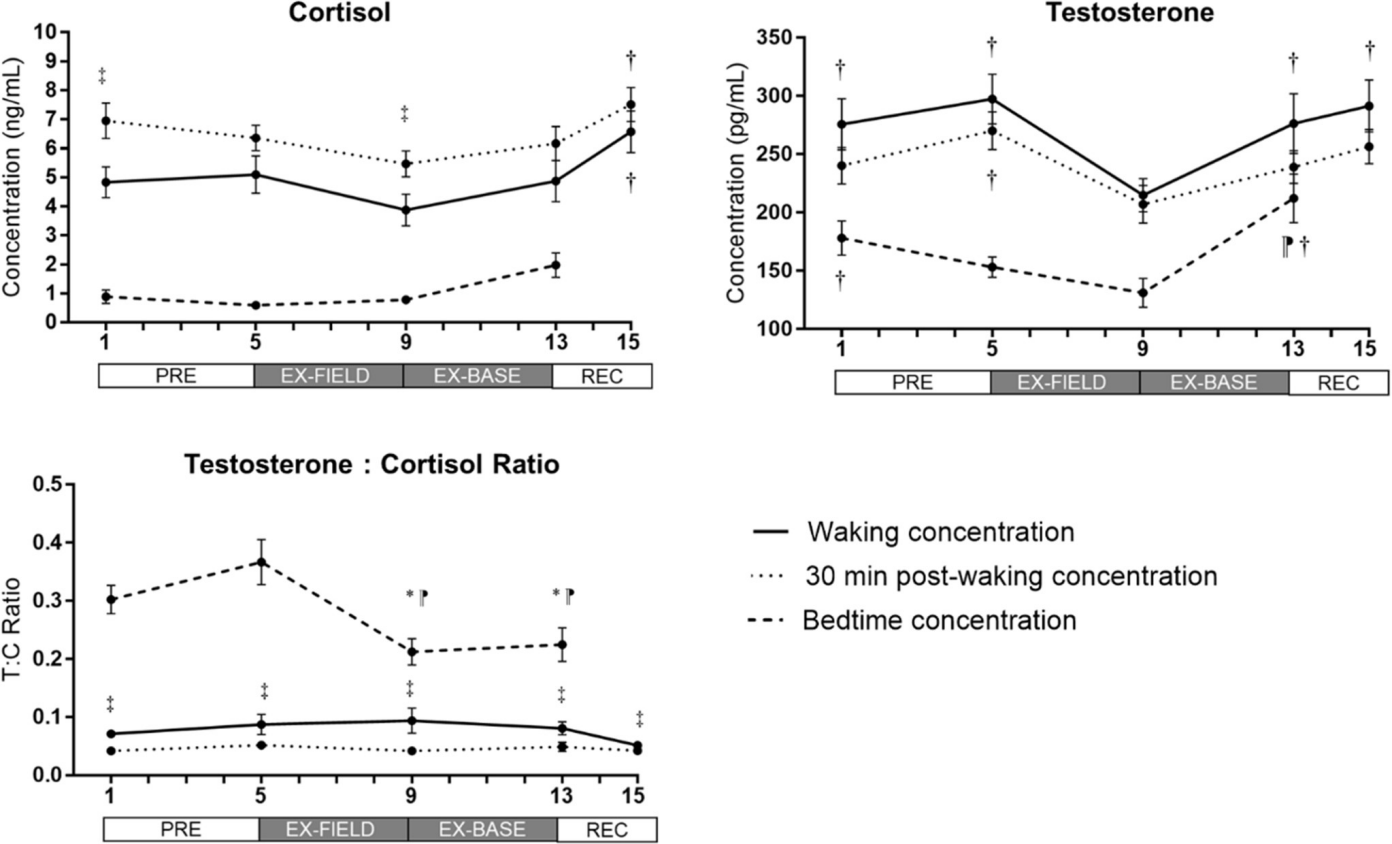
Mean (± SEM) changes in concentrations of salivary cortisol and testosterone, and testosterone/cortisol ratio (T:C ratio) during 16-day study period, including PRE (baseline), EX-FIELD (field training assessment exercise), EX-BASE (simulated base exercise), and REC (recovery period). *p<0.05 vs day 1; ⁋ p<0.05 vs day 5; † p<0.05 vs day 9; ‡ p<0.05 between conditions (waking vs 30 min post-waking).

The cortisol awakening response was no different between days (p = 0.880), with an average CAR of 51.6 ± 14.2% across the training period (mean ± SEM). Across the study period, there was no interaction of condition (diurnal sampling timepoint) and time (p = 0.414). All conditions were significantly different from each other (p<0.001). There were within-group changes in waking (p = 0.013), 30 min post-waking (p = 0.007) and bedtime concentrations (p = 0.001) across the study period (Fig 2). Waking and 30 min concentrations at day 9 were both lower compared to day 15 (69%, p = 0.002 and 37%; p = 0.036 respectively). Concentrations were higher at 30 min post-waking compared to waking, on days 1 and 9 (p<0.020; Fig 2). A selection of participant cortisol responses across the study period, displaying inter-individual variability, are presented in S1 Fig.

#### Testosterone

Across the study period, there was an interaction between condition and time (p = 0.021), and all conditions were significantly different from each other (all p<0.041). There was a within-group difference in waking testosterone concentration (p<0.001) (Fig 2); day 9 concentration was lower than days 1 (28%; p = 0.003), 5 (38%; p<0.001), 13 (28%; p = 0.006), and 15 (35%; p<0.001). There was a within-group change in 30 min post-waking concentration (p = 0.004); lower at day 9, compared to day 5 (30%; p = 0.003). Bedtime concentration changed across the study period (p = 0.002); concentration at day 13 (REC) was higher compared to days 5 (39%; p = 0.024) and 9 (61%; p<0.001), and concentration at day 9 was lower than day 1 (36%;p = 0.042). A selection of participant testosterone responses across the study period, displaying inter-individual variability, are presented in S2 Fig.

#### Testosterone/Cortisol ratio

There was a trend for an interaction between condition and time (p = 0.051). All conditions were significantly different from each other across the study period (p<0.001). Concentration ratios were different between waking and 30 min post-waking at all timepoints (p = 0.038). Waking and 30 min post-waking ratios did not change across the study period (both p>0.137) (Fig 2). Bedtime ratios changed across the study period (p = 0.002; Fig 2). Ratios at day 13 (REC), were lower than days 1 (35%; p = 0.040) and 5 (63%; p = 0.006), and lower at day 9, compared to days 1 (42%; p = 0.035) and 5 (73%; p = 0.002).

### Sleep

Participants averaged 5.8 ± 0.1 h (mean ± SEM) sleep during PRE, 3.9 ± 0.1 h sleep during EX (which included 2.2 ± 0.2 h sleep over the days/nights in FIELD, and 5.8 ± 0.1 h sleep over 4 days/nights at BASE), and 5.9 ± 0.1 h during the first two nights of REC.

### Subjective stress and recovery

**Fig 3:**
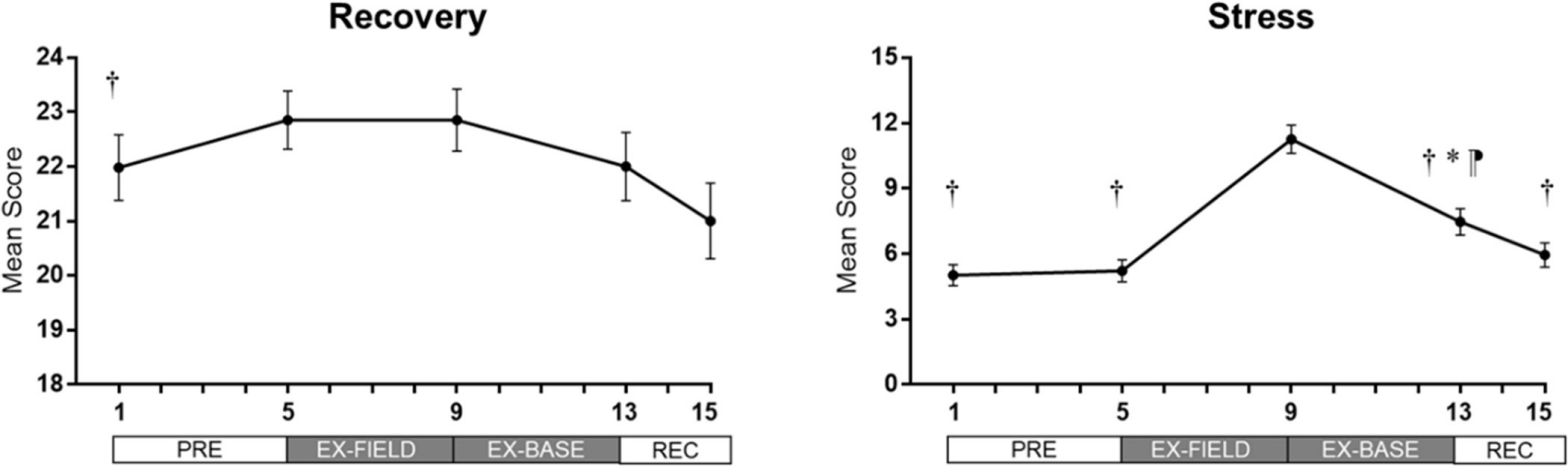
Mean (± SEM) changes in self-reported stress and recovery during 16-day study period. *p<0.05 vs day 1; ⁋ p<0.05 vs day 5; † p<0.05 vs day 9.

Recovery scores changed across the study period (p = 0.013); scores at day 9 were higher than day 1 (p = 0.044; Fig 3). Stress scores underwent within-group changes (p<0.001); higher at day 9 than days 1 (p<0.001), 5 (p<0.001), 13 (p<0.001), and 15 (p<0.001). Stress scores were higher at day 13 compared to days 1 (p = 0.001) and 5 (p = 0.030), with a trend to be higher than day 15 (p = 0.058).

### Physical demands: Step counts

**Fig 4:**
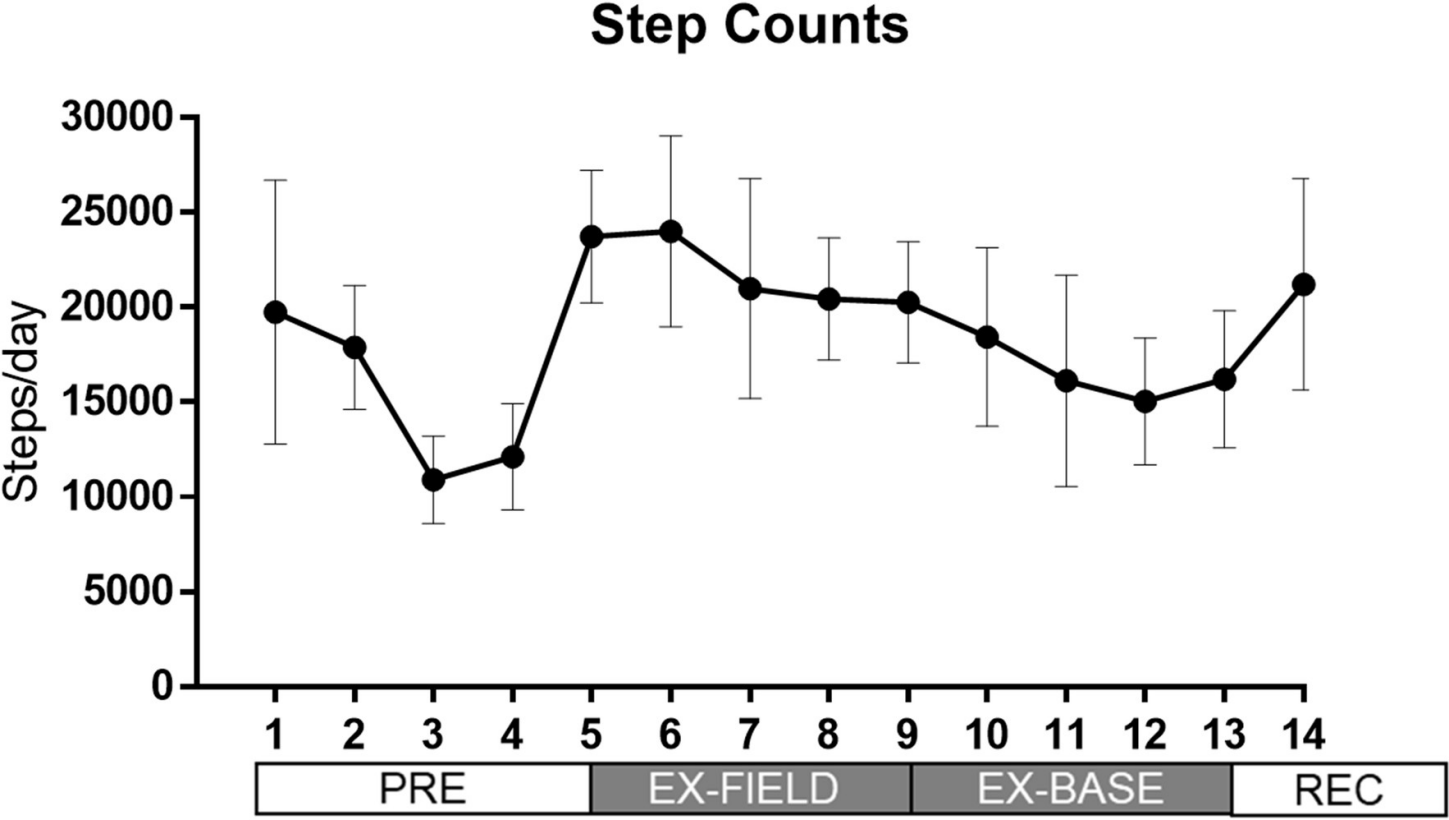
Mean (± SD) changes in daily step count during 16-day study period.

Mean daily step counts changed over the study period (p<0.001; Fig 4). Mean daily step counts were lower during PRE: at day 3 compared to days 1–2, 6–11 and 13–14 (all p<0.01), and lower at day 4 compared to days 1–2, 6–10 and 14 (all p<0.02). Step counts were higher during the initial phase of training (EX-FIELD); at day 5 compared to days 1–4, and 8–13, and higher at day 6 compared to days 1–4, and 7–13 (all p<0.05). Steps taken on days 11, 12 and 13 were lower than days 1, 7–9, and 14 (all p<0.05).

## Discussion

The current study investigated the impact of simulated operational fatigue and reduced sleep on hormonal levels, and the trajectory of hormonal levels over a three-day recovery period post-training. Morning cortisol concentrations were lower following training in the field (day 9) compared to the end of the three-day recovery period. Field training diminished testosterone concentrations, but levels had recovered within four days, and were greater than those before the commencement of field training. Bedtime testosterone/cortisol ratios decreased following training in the field (day 9), and did not return to pre-training levels, indicating a potential disturbance in anabolic/catabolic balance. The period of simulated operational activities was also associated with increased ratings of perceived stress and recovery and increased step counts.

### Cortisol levels during field training exercise

Novel features of our study were that we collected measurements at various timepoints throughout the study period, rather than pre and post measurement, and collected samples at different times of day to capture the influence of circadian variation on hormone levels. Morning salivary cortisol concentrations (waking and 30-min post) were lower following the initial field training phase, compared to the end of the three-day recovery period (69% and 37% respectively). Given that concentrations during the training exercise were not different to baseline (PRE), the exercise did not appear to increase catabolic processes. Our data contrasts with previous training studies that have observed increases in cortisol up to 154% during strenuous periods of training [14,30], that may take in excess of two weeks to recover. However, direct comparison is difficult as these studies incorporated different combinations and/or more challenging stressors such as caloric restriction, live fire drills, greater sleep restriction, extreme amounts of physical activity, and threat of course removal [14,30]. These courses were also designed to teach trainees survival in austere environments, within longer programs. This notion is supported by the average daily step count during the training exercise (mean ~20, 246 steps). Despite being almost twice that recorded in days 3 and 4 before training, this resembles the ~19,000 steps taken daily, on average, by Australian Army recruits during their 10-day field component of basic training (unpublished data). Therefore, the level of physical exertion experienced by trainees in the current study is likely not equivalent to the level of physical exertion encountered in survival training or ‘hell week’ during a special forces selection course [14,30], and in part explains our contrasting findings. Instead, the field training exercises in the current study involved total deprivation and sleep restriction, in combination with intermittent periods of physical activity and cognitive demands. Previous training studies involving training components closer to those contained within the current study have shown no change [40,41], or decreases in cortisol levels after field training, compared to later recovery periods [16,31]. Therefore, cortisol levels during military training appear to be influenced by discrete training elements within a program.

The period of field training involving a combination of total sleep deprivation and severe sleep restriction (EX-FIELD), whereby soldiers averaged 2.2 hours sleep over four nights, is likely to have contributed to the current findings. Prolonged wakefulness (>48 h) is associated with decreased diurnal cortisol levels in laboratory studies, while chronic sleep restriction; <4h sleep for one to six nights has been associated with a lower CAR, lower morning cortisol levels, and increased evening patterns [22,42]. These alterations have been linked to increased physiological stress or perceptions of stress [43]. We did not observe all of these changes to cortisol parameters, however subjective ratings of stress were significantly increased following field training, and only returned to baseline levels at the end of the three-day recovery period. This suggests that observed decreases in morning cortisol may in part be ascribed to higher perceptions of physical and psychological stress, while accumulated sleep loss may also play a role in the disturbance of circadian cortisol release. More frequent sampling between days 5 and 9 of the program may have revealed transient increases following the night of sleep deprivation [22], before a gradual decrease, confirming these associations. Effective responses of the adrenal cortex within multi-stressor operational environments, and also non-stressful situations, is beneficial for soldier performance [44], and long-term health [43,45], and should be targeted in future research.

### Testosterone levels during field training exercise

Our sampling methods attempted to capture the impact of training on circadian timepoints [46,47], and their recovery, rather than a single daily measurement, which has not been attempted in military research, and rarely in field-based research. Our findings indicated that waking and 30 min post-waking total testosterone concentrations decreased by 28% and 23% respectively, over a four-day period, between the start and end of the EX-FIELD component. In addition, bedtime testosterone levels declined by 26% between PRE and the end of EX-FIELD, but had increased by 62% within the next four days. These findings support previous research of strenuous short-term military field training lasting between 5 days and 3 weeks, which have reported 13–47% decreases in serum morning testosterone [13,16,40]. Our study did not impose any caloric restrictions relative to energy expenditure, and therefore higher levels of perceived physical or psychological stress, combined with physical exertion over extended time periods, may explain our lower concentrations of testosterone, as has been previously described [31,48,49]. The timing of the decrease we observed may be explained by anticipatory stress [49], as the upcoming training phase (EX-BASE) included simulated combat against enemy forces. This is reflected in the coincident higher levels of self-reported physical stress, which in conjunction with the training demands induced a greater testosterone usage, and a higher uptake of testosterone at the steroid receptor level [50,51]. It is unclear as to whether paradoxically greater perceptions of recovery at the end of field training reflected an increased uptake of testosterone, or whether soldiers were more aware of their need to engage in recovery processes due to feelings of physical fatigue. Collectively, this suggests a role for subjective measures in combination with objective measures to monitor fatigue and recovery.

Sleep length is crucial for testosterone levels [52], and increasing sleep before periods of deprivation exerts minimal effects on ensuing reductions in testosterone [53], suggesting that diurnal testosterone rhythms are sensitive to sleep loss. Soldiers obtained, on average, 2.2 hours sleep over the EX-FIELD training period, including 40 hours of prolonged wakefulness at the start of the phase. Sleep deficits may have therefore also contributed to declines in testosterone, which supports contentions from previous military studies involving restricted sleep during training (3 to 4 hours per night) [14,16]. Lab-based studies have also associated lower morning testosterone levels with interrupted sleep [54], chronic sleep restriction (8 days of 5 h) [18], and 24–48 h of wakefulness [55,56], due to a reduction in pituitary gonadal axis activity. This line of reasoning is further supported by the observed return of morning testosterone to baseline levels which occurred before the commencement of the actual recovery period, and at the end of a four-day period (EX-BASE) where soldiers accumulated ~6 h sleep per 24 h at a makeshift base, despite still undertaking field manoeuvres. While this duration may still be considered restricted, these opportunities were associated with the restoration of testosterone levels in our study, and previous research [14,53], suggesting the restoration of testosterone levels may be dependent on the provision of sleep, rather than a full reduction in physical load. Given the importance of testosterone in neurocognitive and bone health [57], and positive training adaptations [58], concentrations of this marker may be useful in indicating a soldier’s fatigue and strain [59], recovery and capacity, informing operational readiness [13,14,26].

### Impact of training on testosterone/cortisol ratio and its recovery

The testosterone/cortisol ratio indicates anabolic and catabolic balance, and may be more useful as a potential marker of the physiological strain of training, rather than overtraining [5,25]. In the current study, bedtime testosterone/cortisol ratio was 42% lower at the end of field training component compared to PRE and had not recovered by the evening of day 14. This decrease exceeds the long-standing threshold for overtraining (30%) adopted for athletic populations, the use of which may be outdated [60]. Few military training studies have investigated the response of this ratio, reporting dramatic decreases (up to 87%) that can take between 3 days to one week to resolve [14,31]. Furthermore, recruits classified as “overtrained” and having a high incidence of sickness absence have exhibited lower ratios [7]. As a justification for including the T:C ratio in the current study, the data collection period and training exercise occurred at the end of an 18-week training course. Therefore, it is possible that over the course of the 18 weeks trainees may have been on a trajectory to non-functional overreaching, which precedes overtraining. The decrease of this ratio and reductions in salivary testosterone suggest that the field training component induced a reduction in the anabolic potential of the soldiers, which if prolonged may prevent or slow the recovery of performance [61,62]. However, as cortisol did not concomitantly increase, it is unclear whether a catabolic state was produced. The testosterone/cortisol ratio should be further investigated as a marker of acute and chronic physiological stress in military training.

### Strengths and limitations

There are strengths with this study. To our knowledge, this is one of the few studies to evaluate the impact of total sleep deprivation on hormonal levels within an ecologically valid multi-stressor military environment, while also assessing diurnal variation *and* tracking recovery trajectory. There were some limitations which must be considered when interpreting the results. First, due to the use of different assays and media between studies, we cannot compare absolute levels of salivary hormones to serum levels, however our sampling method may be more practical in reducing participant burden. Second, 90% of the soldiers in the study were men, and the findings may not be generalizable to women. Third, due to study aims, factors known to influence hormonal levels (e.g., sleeping habits, fitness, nutrition) were not included in this study, but it is acknowledged they may have influenced findings [63]. Meals and sleep were regulated between participants, mitigating the potential impact of these factors.

### Conclusion

In conclusion, simulated operational activities involving sleep deprivation and restriction affected hormonal levels, with testosterone and the testosterone/cortisol ratio more sensitive than cortisol. Declines in testosterone were observed after a training period including sleep deprivation, however hormonal disturbance was somewhat restored during a subsequent four-day training period involving greater sleep opportunities. Hormonal responses may serve as surrogate markers of physiological strain during training and be useful in indicating soldier readiness within a larger monitoring system.

## Supporting information

S1 Fig Inter-individual differences in changes of waking concentrations of salivary cortisol during 16-day study period, including PRE (baseline), EX-FIELD (field training assessment exercise), EX-BASE (simulated base exercise), and REC (recovery period). Baseline day ‘0’ not represented in figure. Concentrations are expressed as percentage changes from concentration on day 1 of PRE. A: Increase in cortisol before decline at day 9, B: similar to general trend, C & D: greater decline in EX period before dramatic increase by REC, E: gradual increase in cortisol over study period, F: spike in EX with concentration at REC higher than PRE.
(TIF)

S2 Fig Inter-individual differences in changes of waking concentrations of salivary testosterone during 16-day study period, including PRE (baseline), EX-FIELD (field training assessment exercise), EX-BASE (simulated base exercise), and REC (recovery period). Baseline day ‘0’ not represented in figure. Concentrations are expressed as percentage changes from concentration on day 1 of PRE. A: initial increase in testosterone, decline throughout EX and recovery of levels by end of study period, B: greater fluctuations in testosterone across study period, C & E: similar to general trend, D: decline in testosterone during EX followed by a recovery of testosterone above baseline levels, F: initial increase in testosterone, decline in EX-FIELD and recovery to levels similar to baseline.
(TIF)

## Abbreviations

BMI: body mass index
CAR: cortisol awakening response
CV: coefficient of variation
EX: entire 8-day EX-BARDIA training exercise period
EX-FIELD: 4-day training period out in the field
EX-BASE: 4-day training period at simulated base
h: hour(s)
IET: initial employment training
PRE: 4-day period pre-training
REC: 3-day recover period post-training
SEM: standard error of the mean
SD: standard deviation
T:C ratio: Testosterone to cortisol ratio
