# Supplementary figures and images for: Impact of military training stress on hormone response and recovery
Source: PLoS One. 2022 Mar 10;17(3):e0265121. doi: 10.1371/journal.pone.0265121 (PMC8912193; doi:10.1371/journal.pone.0265121)

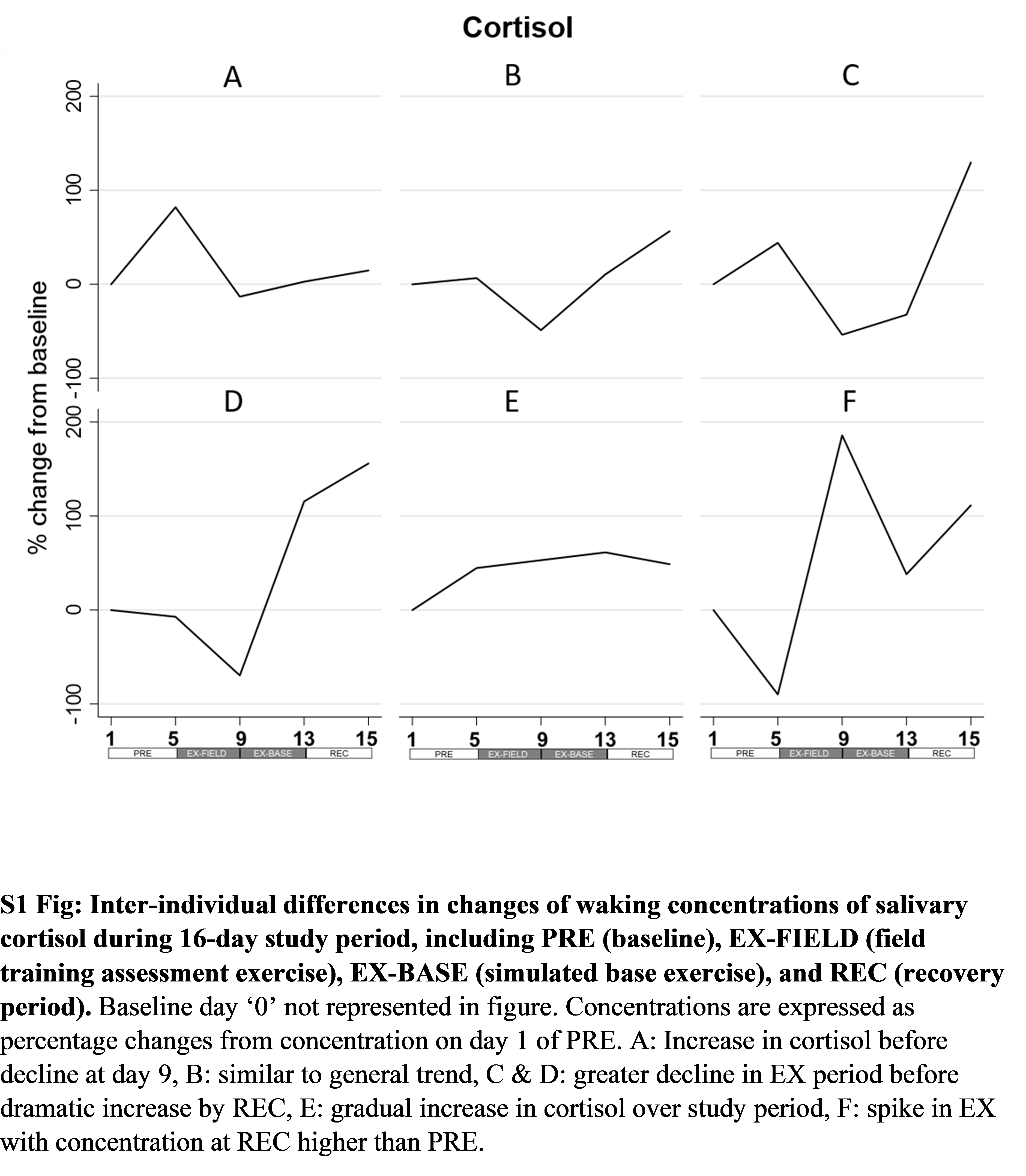

Supplement: S1 Fig — Baseline day ‘0’ not represented in figure. Concentrations are expressed as percentage changes from concentration on day 1 of PRE. A: Increase in cortisol before decline at day 9, B: similar to general trend, C & D: greater decline in EX period before dramatic increase by REC, E: gradual increase in cortisol over study period, F: spike in EX with concentration at REC higher than PRE. (TIF) [file pone.0265121.s001.tif]

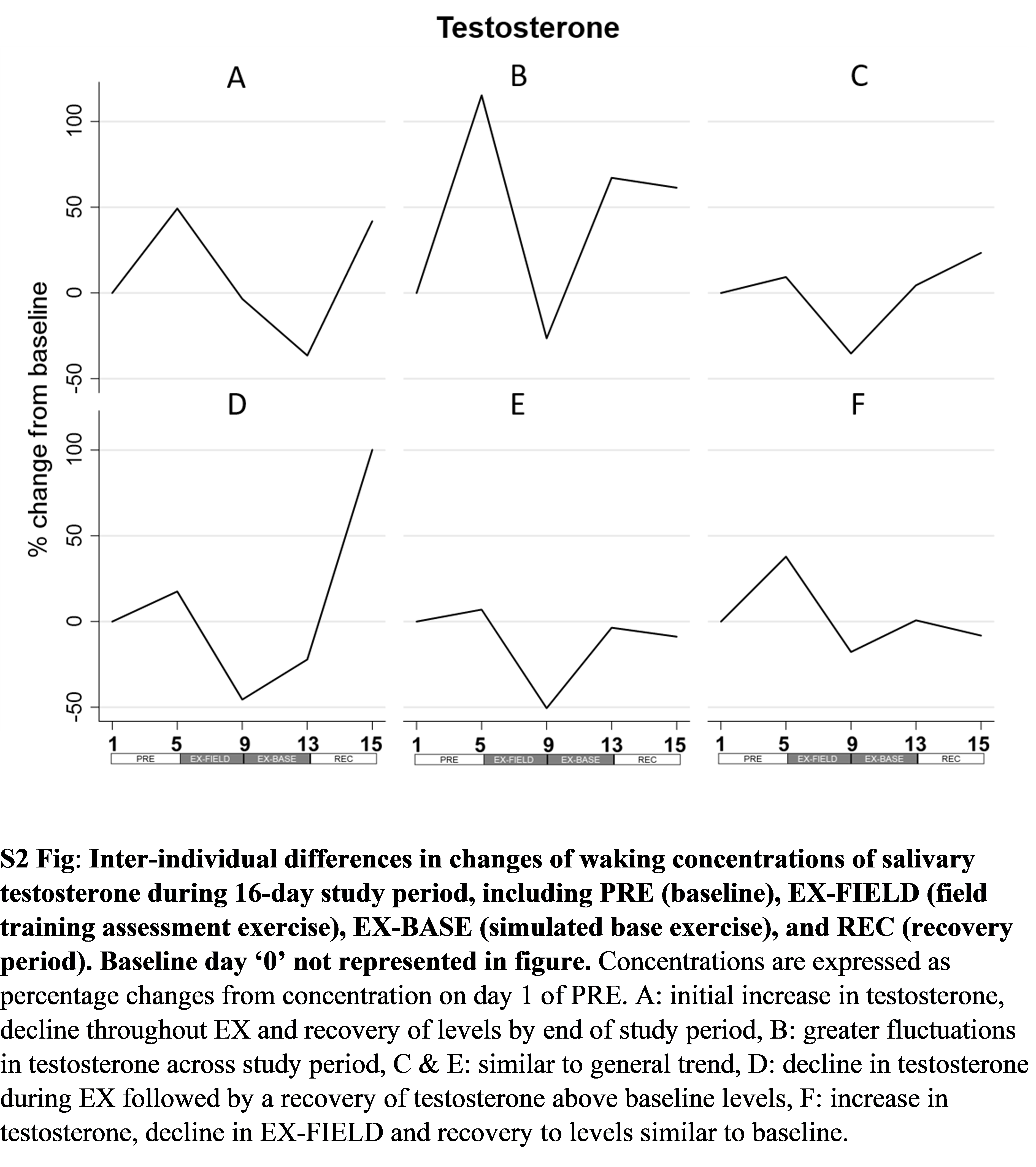

Supplement: S2 Fig — Baseline day ‘0’ not represented in figure. Concentrations are expressed as percentage changes from concentration on day 1 of PRE. A: initial increase in testosterone, decline throughout EX and recovery of levels by end of study period, B: greater fluctuations in testosterone across study period, C & E: similar to general trend, D: decline in testosterone during EX followed by a recovery of testosterone above baseline levels, F: initial increase in testosterone, decline in EX-FIELD and recovery to levels similar to baseline. (TIF) [file pone.0265121.s002.tif]
